# Response and Adaptation of Microbial Community in a CANON Reactor Exposed to an Extreme Alkaline Shock

**DOI:** 10.1155/2020/8888615

**Published:** 2020-06-23

**Authors:** Ruili Yang, Wenlong Mao, Xiaojun Wang, Zhaoji Zhang, Junbin Wu, Shaohua Chen

**Affiliations:** ^1^CAS Key Laboratory of Urban Pollutant Conversion, Institute of Urban Environment, Chinese Academy of Sciences, Xiamen 361021, China; ^2^University of Chinese Academy of Sciences, Beijing 100049, China; ^3^Fujian Agriculture and Forestry University, Fuzhou 350002, China

## Abstract

Responses of a microbial community in the completely autotrophic nitrogen removal over nitrite (CANON) process, which was shocked by a pH of 11.0 for 12 h, were investigated. During the recovery phase, the performance, anaerobic ammonia oxidation (anammox) activity, microbial community, and correlation of bacteria as well as the influencing factors were evaluated synchronously. The performance of the CANON process deteriorated rapidly with a nitrogen removal rate (NRR) of 0.13 kg·m^−3^·d^−1^, and Firmicutes, spore-forming bacteria, were the dominant phyla after alkaline shock. However, it could self-restore within 107 days after undergoing four stages, at which Planctomycetes became dominant with a relative abundance of 64.62%. Network analysis showed that anammox bacteria (*Candidatus Jettenia*, *Kuenenia*, and *Brocadia*) were positively related to some functional bacteria such as *Nitrosomonas*, *SM1A02*, and *Calorithrix*. Canonical correspondence analysis presented a strong correlation between the microbial community and influencing factors during the recovery phase. With the increase of nitrogen loading rate, the decrease of free nitrous acid and the synergistic effects, heme c content, specific anammox activity (SAA), NRR, and the abundance of dominant genus increased correspondingly. The increase of heme c content regulates the quorum sensing system, promotes the secretion of extracellular polymeric substances, and further improves SAA, NRR, and the relative abundance of the dominant genus. This study highlights some implications for the recovery of the CANON reactor after being exposed to an alkaline shock.

## 1. Introduction

A completely autotrophic nitrogen removal over nitrite (CANON) process, a combination of partial nitritation and anaerobic ammonia oxidation (anammox), is highly sensitive to key environmental parameters, such as nitrite (NO_2_^−^-N), dissolved oxygen (DO), and pH [1–3]. Although these parameters are always automatically controlled in engineering applications [4], a fortuitous occurrence of the CANON process can happen accidentally when the automatic control fails. In particular, pH as a crucial parameter can adversely affect the performance of the CANON process if it is not well controlled. In addition, substrate inhibition, which refers to the toxicity of free ammonia (FA) and free nitrous acid (FNA) in the CANON process, is pH-dependent [5]. Therefore, pH can have a strong effect on the CANON process [6]. Research on the effect of alkaline shock on the CANON process often uses pH < 10.0 [7–9]. Fux et al. [10] pointed out that specific anammox activity (SAA) of anammox was completely inhibited by pH 9.3. Li et al. [11] observed the disintegration of anammox granule at pH 9.0. When the concentration of FA reaches approximately 32.5 mg·L^−1^ at pH 8.5, anammox bacteria and ammonia oxidising bacteria (AOB) activities are severely inhibited, and the performance of the CANON system deteriorates quickly [9]. Apart from pH influence, the microbial community is known as an essential factor affecting the efficiency of the CANON process, and the structure of microbial communities is very sensitive to an alkaline situation [12]. In spite of this, studies about the CANON system are mainly focused on the nitrogen removal performance and abundances of anammox bacteria, AOB, and nitrite oxidising bacteria (NOB) [13, 14]. Limited studies have considered the interaction of anammox bacteria, AOB, NOB, and denitrifying bacteria (DNB) [15]. Wu et al. [15] observed that the abundance of *Sphingobacteria*, which is closely related to aerobic denitrifying bacteria, in the CANON system was much higher than that in the anammox system when incubated with anaerobic sludge. Moreover, correlation among the microbial communities in the CANON system in response to alkaline shock remains to be studied in depth. In addition, pH is found to inhibit microbial communities in two ways: (1) pH inhibits the microbial activity by changing the characteristics of microbial habitats [16], and (2) pH inhibits microorganisms directly or via changing the concentrations of FA, FNA, and organics to inactivate irreversible enzymes, obstruct metabolic function, and reduce transcriptional activity [12]. Although the mechanisms of pH effects on the microbial community have been well studied, a few studies have focused on the response of microbial communities during the recovery phase in the CANON process when exposed to alkaline shock.

In this study, we hypothesised that synergy effects among the bacteria associated with the nitrogen cycle would improve the performance of the CANON process after applying transient alkaline pH shock. To test this hypothesis, the CANON process was exposed to an alkaline shock of pH 11.0 for 12 h. The nitrogen removal performance, activity indicators (SAA, heme c, extracellular polymeric substances (EPS), and signalling molecule), variation of the microbial community structure, correlation among the microorganisms, and the correlation between the microbial community and the influencing factors in the CANON reactor were investigated during the whole recovery process. This study is aimed at broadening our current knowledge of the underlying mechanisms of the microbial community in response to an alkaline shock and encouraging technical regulations optimising the CANON process for a maximal efficiency during the recovery process.

## 2. Materials and Methods

### 2.1. Experimental Setup and Reactor Operation

**Figure 1:**
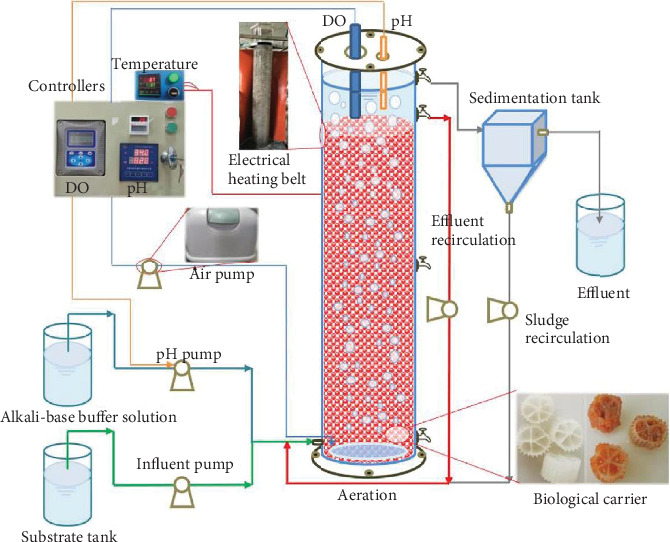
Schematic of the experimental device.

A pilot-scale upflow anaerobic filter (UAF) reactor (Figure 1) was used in this study. It had a working volume of 100 L and an internal diameter of 30 cm and was filled with polypropylene pall rings as biological carriers. A flexible electrical heating belt was adopted to maintain the temperature at 35 ± 1°C and protect anammox bacteria against the competition of photosynthetic microorganisms. The hydraulic retention time (HRT) of the UAF reactor was set at 1 day. The pH and DO were controlled by an online controller and maintained at 7.4–7.8 and 0.02–0.8 mg·L^−1^, respectively [7]. The inoculation sludge was taken from an anammox reactor, and its concentrations of mixed liquor suspended solid (MLSS) and mixed liquor volatile suspended solid (MLVSS) were approximately 22.87 and 12.49 g·L^−1^, respectively.

The CANON process was cultivated using synthetic wastewater, and the mineral medium was supplied according to our previous study [17]. After stable operation for 1.5 y, the CANON process showed a good performance with ammonium removal efficiency and nitrogen removal rate (NRR) of 93.74% and 0.80 kg·m^−3^·d^−1^, respectively. Then, it was exposed to a transient alkaline shock of pH 11.0 for 12 h. The recovery phase mainly consisted of four stages: accommodation stage (days 1–37), anammox recovery stage (days 38–68), CANON recovery stage (days 69–100), and steady stage (days 101–107), according to the nitrogen removal performance of the CANON reactor and a previously reported method by Zhang et al. [18]. The operating conditions of the CANON process during the recovery phase are summarised in Table 1. To ensure the recovery of anammox bacteria activity, first, we inhibited aeration and adjusted the pH to 7.8 by washing bioreactor with the synthetic wastewater without NH_4_^+^-N and NO_2_^−^-N for approximately 1 h at a flow rate of 1600 mL·min^−1^. The influent concentrations of NH_4_^+^-N and NO_2_^−^-N were decreased to 250 mg·L^−1^ to avoid the substrate inhibition [19]. Next, the operating conditions (Table 1) were implemented according to the performance of the CANON reactor.

### 2.2. Sample Collection and Analysis

SAA, an important parameter to evaluate the performance of the anammox pathway [8], and heme c, an indispensable part of the key enzymes [20] directly associated with the substance and energy metabolism of anammox bacteria [21, 22], were used to codetermine the activity of anammox bacteria [23]. EPS, as the stress resister and indicator of the stability and settling property of anammox sludge, coupled with the signalling molecule, are secreted as the metabolites of microorganisms and are beneficial for the formation of biofilm [24, 25]. In this study, EPS was beneficial for initiating the CANON system [26]. The settleability and strength of granule sludge or biofilm are usually evaluated by the ratio of protein (PN) to polysaccharides (PS) [27]. All these factors were used to evaluate the recovery of the CANON process.

Sludge and water samples were withdrawn from the CANON process on days 1, 37, 53, 63, 75, 85, and 107, in order to investigate the EPS concentration, SAA, heme c content, and signalling molecule. Sample collection was conducted in triplicate. Concentrations of EPS and heme c were determined based on the previous reports by Ma et al. [28] and Berry and Trumpower [29], respectively. The semiquantitative estimation of signalling molecule was determined according to the method proposed by Yeon et al. [30]. SAA was conducted according to our previous investigation [31]. Simultaneously, the concentrations of MLSS, MLVSS, ammonia (NH_4_^+^-N), nitrite (NO_2_^−^-N), nitrate (NO_3_^−^-N), and total nitrogen (TN) were analysed based on standard methods [32].

### 2.3. DNA Extraction and Amplicon Sequencing

To investigate responses of microbial communities in the CANON process to the exposure of an alkaline shock, sludge samples collected from the reactor on days 1, 37, 63, and 107 were subjected to DNA extraction, and quality control was also performed according to our previous study [17]. Next, the V3-V4 hypervariable region of 16S rDNA gene of the qualified DNA samples was selected for polymerase chain reaction (PCR) amplification using the universal primer sets: 341F (5′-CCT ACG GGN GGC WGC AG-3′) and 806R (5′-GGA CTA CHV GGG TAT CTA AT-3′) [17]. PCR amplifications were conducted in triplicate, and the products were quantified using a Qubit 2.0 fluorometer (Promega Co., Madison, USA). The purified amplicons were pooled together in an equal amount. Then, the sequencing library was constructed and sent for paired end sequencing on a Hiseq2500 PE250 platform (Gene Denovo Biotechnology Co., Ltd., Guangzhou, China). The sequence data were processed using the Quantitative Insights into Microbial Ecology software [33], and results were assigned to operational taxonomic units (OTUs) with >97% similarity.

### 2.4. Statistical and Network Analyses

Canonical correspondence analysis (CCA) was conducted to further explore the relationship between microbial communities and influencing factors using the “vegan” package in RStudio environment (https://www.rstudio.com/). Pairwise Spearman's correlation coefficients among 12 genera, which have an average abundance higher than 1%, were calculated by SPSS Statistics 22 (IBM, USA) (http://www.ibm.com/analytics/us/en/technology/spss/). Furthermore, network analysis based on Spearman's correlation coefficient (*r*) > 0.6 or <-0.6, and the corresponding *p* value < 0.05 between two genera was performed and visualised by Gephi software (version 0.9.2, https://gephi.org/) [34].

## 3. Results and Discussion

### 3.1. Performance of the CANON Process during the Recovery Phase after Alkaline Shock

**Figure 2:**
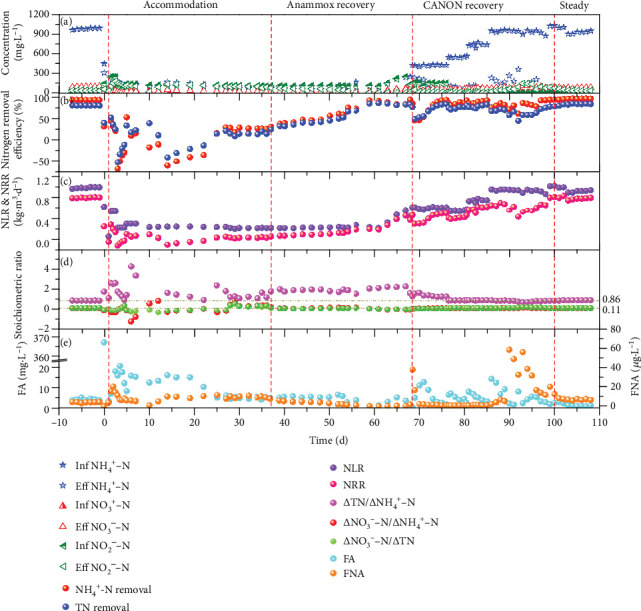
Shifts in nitrogen concentration (a), nitrogen removal efficiency (b), nitrogen loading rate and removal rate (c), stoichiometric ratio (d), and free ammonia and free nitrous acid (e) of the CANON process after alkaline shock.

Figure 2 shows the nitrogen removal performance of the CANON reactor during the experiment. As depicted by Figure 2, the FA concentration of the CANON reactor sharply increased from 3.81 mg·L^−1^ to 367.08 mg·L^−1^, which was much higher than inhibition thresholds of AOB and anammox bacteria after being exposed to alkaline shock of pH 11.0 for 12 h [1, 9]. Thus, the CANON process was severely inhibited by the alkaline shock. As Li et al. [11] reported, the effect of an alkaline pH (9.0) shock on the performance of anammox granular sludge was much more severe than that of an acidic pH (6.5) shock, and the alkaline condition disintegrated anammox granules. Based on the impact of influent pH of 4 and 10 for 12 h, Yu and Jin [35] also found a loss of anammox performance only in alkaline condition. In addition to the increase of FA, researchers suggested that activity loss could be related on the unavailability of trace elements as caused by extreme alkaline condition [35, 36]. Subsequently, aeration, pH, and influent concentrations of NH_4_^+^-N and NO_2_^−^-N were adjusted according to Table 1. Despite this, a progressive deterioration of the nitrogen removal performance was still observed, and the concentration of effluent TN was 41.21% higher than that of influent at the early accommodation stage (days 1–14). However, in the late accommodation stage (days 15–37), the CANON system gradually adapted to the changed environment.

On days 38–52, the nitrogen removal performance of the anammox process recovered slowly with an increase of nitrogen removal efficiency (NRE) from 32% to 49.67%. Afterwards, the NRE and NRR increased rapidly to 86.44% and 0.18 kg·m^−3^·d^−1^, respectively, within 9 days. This phenomenon was similar to the lag stage in the start-up of the anammox process [37]. On days 62–68, the anammox process was enhanced rapidly with NRR increasing from 0.18 to 0.45 kg·m^−3^·d^−1^. To improve the nitrogen removal performance of the CANON system during the recovery stage (days 69–100), DO was maintained at 0.2–0.8 mg·L^−1^. As expected, the NRR increased to 0.82 kg·m^−3^·d^−1^ and NRE was at around 72%. Simultaneously, the stoichiometric ratios of *Δ*TN/*Δ*NH_4_^+^-N, *Δ*NO_3_^−^-N/*Δ*NH_4_^+^-N, and *Δ*NO_3_^−^-N/*Δ*TN gradually increased from 1.29, 0.10, and 0.10, respectively, on day 1, to their theoretical value (0.86, 0.11, and 0.127, respectively), as described in the relevant studies [8, 38]. This meant there was significant synergy between AOB and anammox bacteria and there was no excess proliferation of NOB in the CANON reactor. In addition, the effect of the FNA fluctuation (>15 *μ*g·L^−1^) on nitrogen removal performance was more significant (*p* < 0.05) than that of the FA fluctuation on days 62–68. Previous studies report the FNA inhibition of AOB activity to be 100 *μ*g·L^−1^ [9], whereas the inhibition on anammox bacteria activity is approximately 15 *μ*g·L^−1^ [1]. Deterioration of the CANON performance, which was affected by FNA fluctuation, could be explained by this reason during the recovery phrase of the CANON system. He et al. [1] also pointed out that the anammox pathway, after being exposed to transient pH (9.0) shock, could be recovered rapidly by simultaneously increasing nitrogen loading rate (NLR) and decreasing FNA (<15 *μ*g·L^−1^).

At the steady stage (days 101–107), the water quality of effluent and stoichiometric ratios became stable, and the nitrogen removal performance was even better than the initial level where the averages of NRE and NRR increased to 83.28% and 0.80 kg·m^−3^·d^−1^, respectively. The sensibility, low biomass yield, and slow growth rate of anammox bacteria were the main challenges for the application of the CANON process [8], though autotrophic bacteria of AOB and anammox bacteria were simultaneously grown in a single reactor [39]. Recovery of anammox bacteria should be focused on after the CANON reactor is exposed to an alkaline shock.

### 3.2. Changes of SAA, Heme c, EPS, and Signalling Molecule during the Recovery of the CANON Process after Alkaline Shock

**Figure 3:**
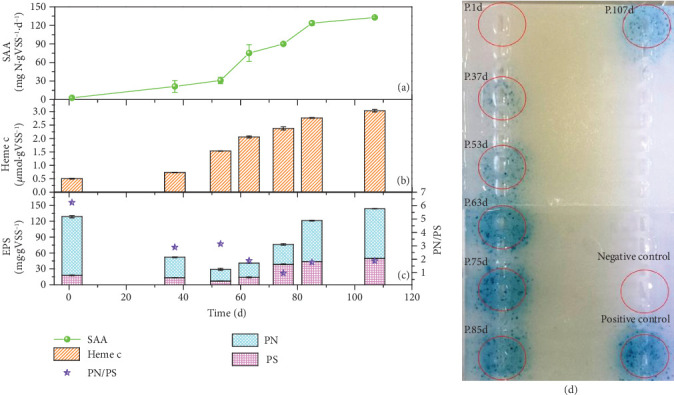
Variations of SAA (a), heme c (b), EPS (c), and signalling molecule (d) content in the CANON process after alkaline shock.

Shifts in SAA, heme c, EPS, and signalling molecule content of the CANON process are shown in Figure 3. The SAA and heme c were maintained at low levels of 2.75 mg N·g^−1^ VSS·d^−1^ and 0.50 *μ*mol·g^−1^ VSS, respectively, on day 1. It was clear that approximately 98% of anammox activity was lost compared to the activity in the initial phase. In the meantime, signalling molecule content was nearly zero, which was the quickest response to the transient alkaline shock. The signalling molecule was easily affected by the pH and quickly degraded under alkaline conditions [40]. It implied that AOB and anammox bacteria were inactivated, which was also supported by the performance of low NRE and NRR on day 1 in the CANON reactor. However, there was a delayed change in EPS, with a high concentration of 128.76 mg·g^−1^ VSS on day 1. This was likely because some microbial members excreted excessive EPS. Subsequently, the signalling molecule-based quorum sensing (QS) system could not regulate EPS production normally after being adversely influenced by the extreme alkaline shock [11, 40]. Previous studies [41, 42] have reported that the signalling molecule-based QS system could regulate EPS secretion under normal conditions. Subsequently, these parameters were gradually recovered during the recovery phase.

Significant increases of SAA, heme c, and the signalling molecule were observed on days 53–63, 37–53, and 53–63, respectively, during which the corresponding NRE were being recovered from 53% to 85%. This meant the anammox bacteria and AOB activities were recovered gradually. Moreover, the recovery time of heme c was shorter than that of the other parameters. Heme c is an important component of hydrazine synthesis, hydroxylamine oxidoreductase, and hydrazine oxidase in purified anammox cells [21]. It is directly associated with the metabolism of substances and energy in anammox bacteria [21, 22], and it also determines the activity of anammox biomass [23]. Chen et al. [43] found a similar result in an anammox-EGSB recovery system which experienced a longer-term starvation. Badalamenti et al. [44] reviewed one of 38 putative multiheme c-type cytochromes containing 69 heme-binding motifs and concluded that a LuxI/LuxR QS cassette can produce an unidentified signalling molecule. A signalling molecule-based QS system could regulate EPS secretion [41, 42]. Zhang et al. [45] confirmed that C8-HSL and C6-HSL, the examples of signalling molecules, favour EPS production and improve the activity of anammox bacteria, AOB, and others. Therefore, the enhancement of heme c was found to accelerate the secretion of a signalling molecule and promote the microbial activity [21, 44]. As a result, an increase in EPS content was found following the changing pattern of the signalling molecule. On day 107, the concentrations of SAA, heme c, and EPS were increased to 132.86 mg N·g^−1^ VSS·d^−1^, 3.03 *μ*mol·g^−1^ VSS, and 143.65 mg·g^−1^ VSS, respectively. Although these values were similar to the findings in previous studies [45, 46], they were much higher than the values at the initial levels (*p* < 0.01) after the alkaline shock in this study. The NLR, NRR, and NRE reached the maximum values, and the CANON system remained stable.

### 3.3. Microbial Community Shift in the CANON Process after Alkaline Shock

**Figure 4:**
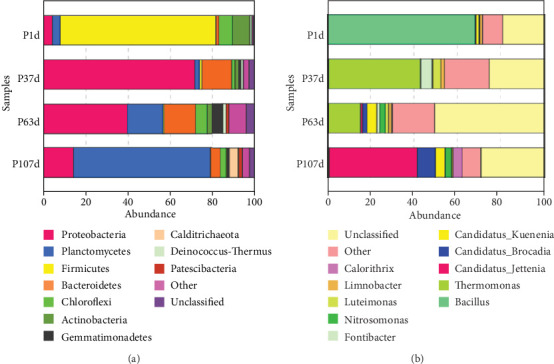
Bacterial community composition at the phylum (a) and genus (b) levels in the CANON process after alkaline shock.

A shift in the microbial community structure of the CANON process after alkaline shock was investigated by amplicon sequencing. The OTU number and diversity index increased gradually as the CANON process resumed (Table 2).  Figure 4(a) shows the bacterial community composition in the CANON process at the phylum level. As shown in Figure 4(a), Firmicutes was the dominant bacterial taxum with a relative abundance of 73.93% after the alkaline shock. A similar phenomenon was also observed by Callejas et al. [47] after a transient pH increase. Wang and Gu [48] reported that the effect of alkaline condition (9.0) on the microbial community structure of the anammox process was stronger than that of acid condition (5.0). Firmicutes are spore-forming bacteria and related to complex organic matter degradation, which could explain the resistance to cell lysis in an alkaline environment [47]. The recovery of the CANON process also experienced four stages according to the variations in the microbial community at the phylum's level. Proteobacteria and Bacteroidetes, which are involved in nitrogen removal and organic acid degradation [49], became the two dominant phyla with their relative abundances increasing from 4.33% to 71.63% and from 1.32% to 14.29%, respectively, at the accommodation stage. Proteobacteria, Planctomycetes, and Bacteroidetes were the three dominant phyla at the growth stage, and the relative abundance of Planctomycetes increased from 2.28% to 16.87%. On day 107, Planctomycetes became the dominant phyla with a relative abundance of 64.62%, indicating that the CANON process had recovered to a certain degree. Wang et al. [50] also observed this phenomenon during the recovery of the CANON process when stressed by salt.

At the genus level (Figure 4(b)), *Bacillus*, belonging to Firmicutes, was the dominant genus with a relative abundance of 67.98% after the alkaline shock. *Bacillus* is a robust model organism for biofilm formation [51]. *Bacillus* can not only contribute to O_2_ removal [52], nitrate reduction [53], and organic matter degradation [54] but can also secrete EPS, especially EPS-PN, for increasing resistances to adverse environmental stresses [55]. All these factors may explain why the *Bacillus* survived after alkaline shock. The accommodation stage (days 1–37) in this study was analogous to the cell lysis stage in the start-up of the anammox process [56]. *Thermomonas* was capable of utilising the organic products from cell lysis [57]; therefore, it became the dominant genus at the accommodation stage. At the growth stage (days 38–61), the microbial diversity of the CANON process was consistently increasing. The relative abundances of anammox bacteria (*Candidatus Jettenia*, *Kuenenia*, and *Brocadia*) gradually increased from 0.13% to 7.52%. After that, AOB (*Nitrosomonas*) together with anammox bacteria (*Candidatus Jettenia*, *Kuenenia* and *Brocadia*) became the dominant genera as aeration began. The relative abundances of AOB and anammox bacteria (*Candidatus Jettenia+Kuenenia*+*Brocadia*) at the enhancement stage increased quickly from 2.57% and 7.52% to 3.05% and 53.30%, respectively. Excess proliferation of NOB was not detected throughout the experiment. The good performance of the CANON process could be explained through microbiological perspectives.

### 3.4. Correlations of Microbial Communities and the Influencing Factors in the CANON Process after Alkaline Shock

**Figure 5:**
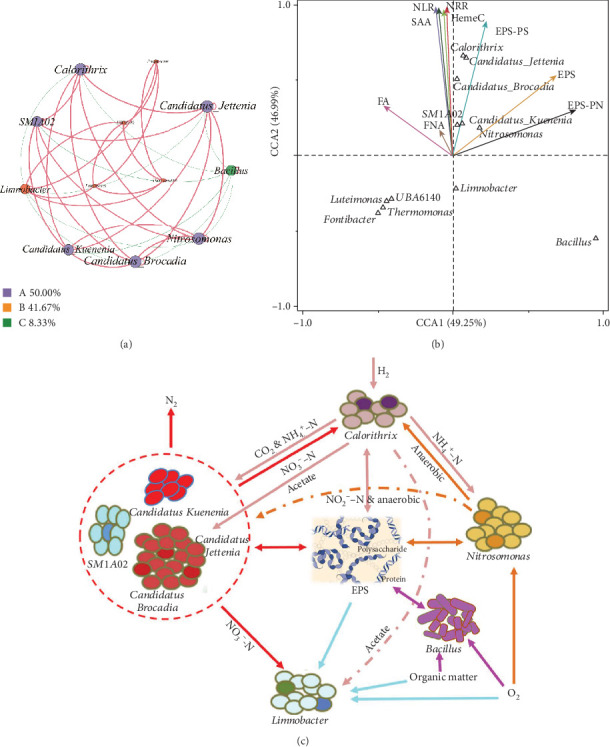
(a) Cooccurring network analysis of dominant microflora, (b) CCA of microbial communities and the influencing factors, and (c) correlation among dominant microflora, in the CANON process exposed to alkaline shock. The cooccurring network is coloured by the modularity class. Each node represents a dominant genus. Red and green lines represent positive and negative correlations, respectively. Node sizes are weighted by the node degree (numbers of connections).

Network analysis is an effective analytical approach for further exploring the correlation among the dominant microbial taxa (Figure 5(a)) in the CANON process exposed to alkaline shock. As shown in Figure 5(a), the dominant flora was divided into three modules based on the Fruchterman-Reingold algorithm. Members in module A, which accounted for 50% of the network, were *Candidatus Jettenia*, *Candidatus Kuenenia*, *Candidatus Brocadia*, *Nitrosomonas*, *SM1A02*, and *Calorithrix*. They were positively related to each other (*p* < 0.05). *Limnobacter*, one genus of module B, showed a significant negative correlation with *Candidatus Jettenia*, *Candidatus Brocadia*, *Nitrosomonas*, and *Calorithrix* (*p* < 0.05). Additionally, *Bacillus* was negatively correlated with all members in module A. CCA was further applied to determine the correlation of microbial communities and the influencing factors (Figure 5(b)) during the recovery phase. As is presented in Figure 5(b), the dominant genera of *Candidatus jettenia*, *Candidatus Kuenenia*, *Candidatus Brocadia*, *SM1A02*, and *Calorithrix*, had a significantly positive correlation (*p* < 0.05) with the influencing factors of SAA, NLR, NRR, heme c, EPS-PS, and FNA. *Nitrosomonas* was positively correlated with EPS, EPS-PN, SAA, NLR, NRR, heme c, EPS-PS, and FNA. However, *Bacillus* was only positively correlated with EPS-PN.

The abovementioned results can be explained by (1) the impact of NLR-led influencing factors, (2) synergistic effects of the dominant genus in the network module A, and (3) antagonistic effect of *Limnobacter*, *Bacillus*, and the dominant genus in network module A. As shown in Figure 5(c), anammox bacteria (*Candidatus Jettenia*, *Brocadia*, and *Kuenenia*) oxidised NH_4_^+^-N to N_2_ with NO_2_^−^-N and CO_2_, which were produced by AOB (*Nitrosomonas*) and DNB (*Calorithrix*), respectively. These served as the electron acceptors and inorganic carbon sources under anaerobic conditions [49, 58]. As reported in previous studies, *SM1A02* has the anammox ability and might be a novel anammox strain; therefore, it has the same synergistic effects with *Nitrosomonas* and *Calorithrix* [59–61]. In addition, *Nitrosomonas* can utilise NH_4_^+^-N and DO and create anaerobic conditions for anammox bacteria such as *SM1A02* and *Calorithrix* [49, 60]. *Calorithrix* provides a substrate of NH_4_^+^-N to anammox bacteria, *SM1A02*, and *Nitrosomonas* by dissimilatory NO_3_^−^-N reduction to NH_4_^+^-N [58]. All members in module A could secrete EPS to protect the members from adverse environmental stresses [58–60]. With the increase of NLR, decrease of FNA, and enhancement of synergistic effects of the dominant genus on network module A, it was observed that heme c content, SAA, NRR, and the relative abundance of dominant genus increased correspondingly [1, 21, 44, 45]. He et al. [1] reported that the anammox reactor recovered rapidly by simultaneously increasing NLR and decreasing FNA after experiencing a transient pH shock. However, *Limnobacter* competed with other functional bacteria for acetate, O_2_, and NO_3_^−^-N [62] and consumed the EPS-PN produced by the members in module A. This explains why *Limnobacter* deteriorated the resistance of the microbial community to adverse influences [62, 63] and was assigned to module B. In addition, *Bacillus* was eliminated in long-term anaerobic conditions with the increase of NLR and therefore was negatively correlated with module A [64]. The synergistic and antagonistic effects of these functional bacteria, coupled with the control of NLR, FA, and FNA, codetermined the performance of the CANON process. All these supplied the underlying mechanisms of the microbial community in response to the alkaline shock. Meanwhile, these could also inform technical regulations optimising the CANON process for maximal efficiency during the recovery process.

## 4. Conclusions

The CANON process was found to rapidly deteriorate after an alkaline shock of pH 11. However, it could be self-restored with the final NRR of 0.80 kg·m^−3^·d^−1^ after the system underwent four stages (accommodation, anammox recovery, CANON recovery, and steady stages). During the entire recovery process, heme c content responded the fastest to the transient alkaline shock among all tested parameters and increased gradually, followed by the signalling molecule. Both parameters improved the AOB and anammox activity and regulated the EPS secretion. Firmicutes, a type of spore-forming bacterium, was dominant at the initial stage, and subsequently at the later period, Planctomycetes became the dominant phylum with a relative abundance of 64.62%. Network analysis clearly revealed positive relationships among anammox bacteria (*Candidatus Jettenia*, *Kuenenia*, and *Brocadia*), AOB (*Nitrosomonas*), *SM1A02*, and DNB (*Calorithrix*). Additionally, there was a strong correlation between microbial communities and the influencing factors during the recovery phase. With the increase of NLR, decrease of FNA, and synergistic effects of dominant microbial members, heme c content, SAA, NRR, and the relative abundance of the dominant genus increased correspondingly. The increase of heme c content regulates the quorum sensing system, promotes the secretion of EPS, and further improves SAA, NRR, and the relative abundance of the dominant genus. The response of the microbial community to environmental change gives an insight into the recovery of the CANON process after being exposed to an alkaline shock and provides technical regulations for its engineering application.

## Figures and Tables

**Table 1:** Operational conditions of the CANON process after the alkaline shock.

Stage	Time (d)	NH_4_^+^-N (mg·L^−1^)	NO_2_^−^-N (mg·L^−1^)	NLR (kg·m^−3^·d^−1^)	DO (mg·L^−1^)
Accommodation stage	1-3	250	250	0.5	<0.05
	4-37	100	100	0.2	
Anammox recovery stage	38-61	100	100	0.2	
	62-64	150	150	0.3	
	65-68	250	250	0.5	
CANON recovery stage	69-76	450	150	0.6	0.2-0.8
	77-81	600	0	0.6	
	82-86	800	0	0.8	
	87-100	1000	0	1	
Steady stage	101-107	1000	0	1	

**Table 2:** Characteristics of amplicon libraries.

Group	OTUs	Shannon	Simpson	Chao	Ace	Coverage
P1d	758	5.04	0.912	812.0	854.41	0.998
P37d	780	4.81	0.829	842.4	884.32	0.998
P63d	1058	6.90	0.973	1096.8	1149.77	0.997
P107d	1063	5.15	0.843	1099.1	1152.35	0.998

## Data Availability

The data used to support the findings of this study are included within the article.
